# Supplementary material for: ONC201 and an MEK Inhibitor Trametinib Synergistically Inhibit the Growth of Triple-Negative Breast Cancer Cells
Source: Biomedicines. 2021 Oct 7;9(10):1410. doi: 10.3390/biomedicines9101410 (PMC8533320; doi:10.3390/biomedicines9101410)
Supplement: Supplementary file 1 [file biomedicines-09-01410-s001.zip › biomedicines-1361838-supplementary.pdf]

Table S1. The top 100 target kinases identified in CAL51 TNBC cell line using 3D RNAi kinome-wide library screening.

CAL51													
**Rank**	**Gene Symbol**	**Rank**	**Gene Symbol**	**Rank**	**Gene Symbol**	**Rank**	**Gene Symbol**	**Rank**	**Gene Symbol**	**Rank**	**Gene Symbol**	**Rank**	**Gene Symbol**
1	*RAC1*	35	*LYN*	69	*GUK1*	103	*PIK3R4*	137	*PRKCL2*	171	*MERTK*	205	ACVRL1
2	*MAP4K4*	36	*IKBKE*	70	*PIK4CA*	104	*PCTK3*	138	*NME4*	172	*MAPK14*	206	C5R1
3	*RASGRF2*	37	*EPHB1*	71	*MAPK6*	105	*AVPR1B*	139	*NEK4*	173	*RPS6KA4*	207	MAP2K4
4	*FUK*	38	*IL2*	72	*SNF1LK*	106	*CCL3*	140	*ITPK1*	174	*EEF2K*	208	PTPRT
5	*GK2*	39	*PIM1*	73	*DUSP2*	107	*GNAI1*	141	*HIPK3*	175	*BAI3*	209	MAPKAPK3
6	*ILK*	40	*PRKWNK1*	74	*RAGE*	108	*STK33*	142	*FGFR1*	176	*EDG5*	210	GNAI3
7	*CARKL*	41	*PINK1*	75	*EPHA2*	109	*MAP4K2*	143	*PIP5K1A*	177	*TRIM*	211	SNARK
8	*PRKCN*	42	*PIK3R1*	76	*GNB4*	110	*ADRBK1*	144	*HK2*	178	*NME7*	212	TPK1
9	*HIPK2*	43	*LIM*	77	*CXCL2*	111	*HK3*	145	*PLK3*	179	*MAP3K2*	213	GHRHR
10	*EPHB6*	44	*EPHB3*	78	*MGC5601*	112	*PIK3CB*	146	*C14ORF20*	180	*PHKG1*	214	CDK5RAP3
11	*CDC2L1*	45	*HUNK*	79	*PXK*	113	*LMTK3*	147	*MOS*	181	*PPP2CA*	215	MELK
12	*PKN3*	46	*EPHA5*	80	*STK17A*	114	*MYO3B*	148	*MAPK12*	182	*PTK9*	216	STK29
13	*HSMDPKIN*	47	*MAP2K5*	81	*MPP3*	115	*CDKN2B*	149	*PANK1*	183	*PFKFB2*	217	TLK2
14	*CDKN3*	48	*MARK1*	82	*HK1*	116	*PRKAA1*	150	*MYO3A*	184	*PRKAR2A*	218	NBEA
15	*EPHB2*	49	*MPZL1*	83	*PRKCM*	117	*CHUK*	151	*PTPRG*	185	*MAPKAPK2*	219	FZD5
16	*PRPSAP2*	50	*KIF13B*	84	*NME6*	118	*PAK6*	152	*APPL*	186	*TAO1*	220	IRS1
17	*MGC8407*	51	*PRKCABP*	85	*STK32B*	119	*CHRM1*	153	*FZD7*	187	*EDG6*	221	STK6
18	*GSK3B*	52	*MAP4K3*	86	*CALCA*	120	*PAK4*	154	*EPHA3*	188	*CCR6*	222	GNAT2
19	*NAGK*	53	*PDGFRA*	87	*MAPK13*	121	*KDR*	155	*HIPK4*	189	*PIK4CB*	223	EDNRA
20	*FLJ13052*	54	*NEK6*	88	*GNB1*	122	*IKBKB*	156	*PIK3R3*	190	*TXNDC3*	224	ADRA2A
21	*NPR1*	55	*PRKCA*	89	*PRKAR2B*	123	*CDKN2C*	157	*CX3CR1*	191	*ASP*	225	BMPR1A
22	*ITGB1BP1*	56	*PTK9L*	90	*PIK3R2*	124	*CDKN2D*	158	*MAPK4*	192	*RIPK2*	226	BLK

23	*MGC16169*	57	*MAP3K3*	91	*F2R*	125	*F2RL2*	159	*MAP3K14*	193	*CSNK1G3*	227	NEK3
24	*PFKFB3*	58	*MRC2*	92	*GNA14*	126	*PKMYT1*	160	*BAI2*	194	*EBI2*	228	NEK2
25	*PIP5K2A*	59	*EPHA8*	93	*PIK3CG*	127	*CNR1*	161	*CKMT1*	195	*EDG4*	229	ASK
26	*NEK8*	60	*PYCS*	94	*BDKRB1*	128	*GNG11*	162	*OSR1*	196	*INSRR*	230	CRHR2
27	*FLJ34389*	61	*EPHA4*	95	*AKT3*	129	*TEX14*	163	*C9ORF98*	197	*DRD1*	231	MAP3K13
28	*CAMK2A*	62	*CDKL1*	96	*GSK3A*	130	*FRK*	164	*PKM2*	198	*MAPK3*	232	FGR
29	*PTPN5*	63	*PACE-1*	97	*MAP2K3*	131	*SCYL1*	165	*PTPRR*	199	*SCAP1*	233	AGTRL1
30	*CDK4*	64	*TNIK*	98	*PRPSAP1*	132	*DAPK2*	166	*TP53RK*	200	*MAP3K8*		
31	*PIM2*	65	*EPHB4*	99	*IHPK3*	133	*PRKCI*	167	*DRD5*	201	*PDXK*		
32	*PANK3*	66	*PRKAA2*	100	*PRPS2*	134	*EPHA7*	168	*PTPRJ*	202	*MAP3K11*		
33	*BMPR1B*	67	*RAF1*	101	*DGKG*	135	*NEK7*	169	*CDKL5*	203	*SQSTM1*		
34	*DLG1*	68	*F2RL1*	102	*CLK2*	136	*PIK3CA*	170	*CCRL1*	204	*IHPK2*		

Table S2. The top 100 target kinases identified in HCC70 TNBC cell line using 3D RNAi kinome-wide library screening.

HCC70															
**Rank**	**Gene Symbol**	**Rank**	**Gene Symbol**	**Rank**	**Gene Symbol**	**Rank**	**Gene Symbol**	**Rank**	**Gene Symbol**	**Rank**	**Gene Symbol**	**Rank**	**Gene Symbol**	**Rank**	**Gene Symbol**
1	*DGKB*	36	*FGR*	71	*HCK*	106	*CSNK1A1L*	141	*MAPK6*	176	*DUSP8*	211	*GSK3A*	246	*GPR155*
2	*CELSR2*	37	*BCKDK*	72	*SCYL1*	107	*CAMK1D*	142	*DLG4*	177	*MARK2*	212	*AATK*	247	*RET*
3	*MASTL*	38	*STK23*	73	*TTK*	108	*FN3KRP*	143	*DKFZP761P0423*	178	*FRDA*	213	*PDXK*	248	*CDKN2C*
4	*GIPR*	39	*CLK3*	74	*EPHA5*	109	*CDKN1B*	144	*GNAO1*	179	*KHK*	214	*ERBB3*	249	*TTN*
5	*URKL1*	40	*CASK*	75	*PFTK1*	110	*MGC16169*	145	*ADRBK2*	180	*CCRL2*	215	*TPK1*	250	*TOPK*
6	*DGKZ*	41	*DGKA*	76	*GUK1*	111	*FLJ10458*	146	*CDADC1*	181	*LMTK2*	216	*LIMK1*	251	*PIM2*

7	*DUSP10*	42	*PLK2*	77	*MGC5601*	112	*PACE-1*	147	*FLJ10060*	182	*AURKC*	217	*GLP2R*	252	*IRS1*
8	*BRAF*	43	*CAMK1*	78	*EPHA1*	113	*FER*	148	*FZD9*	183	*FPR1*	218	*DYRK3*	253	*RNASEL*
9	*DGKE*	44	*PGK1*	79	*APEG1*	114	*CSNK2A1*	149	*TTBK1*	184	*CDK8*	219	*PCTK3*	254	*FLJ25006*
10	*BAI2*	45	*ADRB2*	80	*ARK5*	115	*CSNK1G3*	150	*KIT*	185	*C3AR1*	220	*GNB4*	255	*IKBKE*
11	*EPHB1*	46	*ITK*	81	*DUSP22*	116	*TNNI3K*	151	*BMX*	186	*PAPSS1*	221	*RP2*	256	*EGFR*
12	*DUSP4*	47	*DDR1*	82	*FKSG83*	117	*GRK6*	152	*PCTK2*	187	*CDKN2D*	222	*IGF1R*	257	*PIM1*
13	*FLJ32685*	48	*LIM*	83	*GFRA2*	118	*CCR2*	153	*ULK1*	188	*ROCK2*	223	*C5*	258	*TGFBR2*
14	*MAP2K1IP1*	49	*CDKN1C*	84	*PRPS1*	119	*TGFBR1*	154	*CALCRL*	189	*BLR1*	224	*TESK2*	259	*MAPK3*
15	*CDK5R2*	50	*RFP*	85	*CAMK2D*	120	*CHUK*	155	*DEFB4*	190	*EDG5*	225	*ARRB1*	260	*CDC2*
16	*BMPR2*	51	*ICK*	86	*HIPK2*	121	*IKBKB*	156	*ADRB2*	191	*EPHB3*	226	*ELOVL4*	261	*CDKN3*
17	*AVPR1B*	52	*MAP3K2*	87	*FZD6*	122	*GNAT1*	157	*EBI2*	192	*CINP*	227	*EDG8*	262	*PFKFB4*
18	*FLJ10842*	53	*MGC8407*	88	*DYRK1B*	123	*PRKCABP*	158	*APPL*	193	*ERBB4*	228	*ACVRL1*	263	*CDK5*
19	*RPS6KA5*	54	*CD7*	89	*FGFR2*	124	*CYSLTR1*	159	*PRKCQ*	194	*C7ORF16*	229	*CXCL12*	264	*ACVR1C*
20	*STK38L*	55	*KIF13B*	90	*CDC7*	125	*KSR2*	160	*AAK1*	195	*RAGE*	230	*CCR1*	265	*DLG1*
21	*ANKRD3*	56	*AVPR1A*	91	*CASR*	126	*GTF2H1*	161	*CDC42BPB*	196	*DGUOK*	231	*CARD10*	266	*PRKCH*
22	*ADRA1B*	57	*AK3L1*	92	*MAP4K4*	127	*MST4*	162	*GUCY2F*	197	*LOC91807*	232	*PI4KII*	267	*F2RL1*
23	*VRK2*	58	*MPZL1*	93	*ARAF1*	128	*AKT3*	163	*PDK3*	198	*SGKL*	233	*GNAS*	268	*NYD-SP25*
24	*GNG11*	59	*MGC4796*	94	*RIPK1*	129	*AGTR2*	164	*CHRM3*	199	*DYRK4*	234	*CAMK2A*	269	*RPS6KB1*
25	*CCL17*	60	*C5R1*	95	*CALCR*	130	*TAF1*	165	*FUK*	200	*BAI1*	235	*KIAA1399*	270	*RPS6KC1*
26	*BMPR1B*	61	*FZD5*	96	*C17ORF35*	131	*STK24*	166	*CSNK1G1*	201	*CAMK2G*	236	*KDR*	271	*FLJ35107*
27	*BMP2K*	62	*GIT2*	97	*SMAD7*	132	*DGKD*	167	*CCL2*	202	*RYK*	237	*DUSP1*	272	*PRPSAP1*
28	*CRY1*	63	*CARD14*	98	*ADORA3*	133	*EDG4*	168	*SNARK*	203	*TK2*	238	*CCR8*	273	*BRS3*

29	*LOC115704*	64	*SOCS1*	99	*HRI*	134	*ADCYAP1R1*	169	*PRPS2*	204	*PFKFB1*	239	*DLG3*	274	*PAK6*
30	*ICF45*	65	*EEF2K*	100	*PRKDC*	135	*PDK4*	170	*FASTK*	205	*FPRL2*	240	*GMFB*	275	*IRAK2*
31	*CSF1R*	66	*EDG3*	101	*FRAP1*	136	*CXCL10*	171	*CXCL9*	206	*TRIO*	241	*CNKSR1*	276	*SAST*
32	*DGKG*	67	*MPP2*	102	*LTK*	137	*SGK*	172	*BUB1B*	207	*PIK3CA*	242	*ADORA2B*	277	*STYK1*
33	*GCK*	68	*CXCL3*	103	*KIS*	138	*MAP4K3*	173	*ADK*	208	*GNAQ*	243	*ILKAP*	278	*PRKWNK2*
34	*AKAP1*	69	*BCR*	104	*EPHA8*	139	*RIPK2*	174	*CAMK4*	209	*SHC1*	244	*CRHR2*	279	*EDG1*
35	*TLR3*	70	*CDKL1*	105	*PRKACB*	140	*CCL3*	175	*ADORA1*	210	*PFKL*	245	*C7ORF9*		

Table S3. The 65 common target genes from CAL51 and HCC70 using 3D RNAi kinome-wide library screening.

**65 Common Target Genes**									
ACVRL1	*AKT3*	*APPL*	*AVPR1B*	*BAI2*	*BMPR1B*	*C5R1*	*CAMK2A*	*CCL3*	*CDKL1*
CDKN2C	*CDKN2D*	*CDKN3*	*CHUK*	*CRHR2*	*CSNK1G3*	*DGKG*	*DLG1*	*EBI2*	*EDG4*
EDG5	*EEF2K*	*EPHA5*	*EPHA8*	*EPHB1*	*EPHB3*	*F2RL1*	*FGR*	*FUK*	*FZD5*
GNB4	*GNG11*	*GSK3A*	*GUK1*	*HIPK2*	*IKBKB*	*IKBKE*	*IRS1*	*KDR*	*KIF13B*
LIM	*MAP3K2*	*MAP4K3*	*MAP4K4*	*MAPK3*	*MAPK6*	*MGC16169*	*MGC5601*	*MGC8407*	*MPZL1*
PACE-1	*PAK6*	*PCTK3*	*PDXK*	*PIK3CA*	*PIM1*	*PIM2*	*PRKCABP*	*PRPS2*	*PRPSAP1*
RAGE	*RIPK2*	*SCYL1*	*SNARK*	*TPK1*					

Table S4. The 24 AS-related proteins used in RPPA analysis.

**Apoptosis Related 24 Proteins from RPPA Analysis**			
H2AX pS140	FoxO3 pS318	Annexin I	PARP
Bcl2	Bcl-XL	Bax	Wee1
Caspase 8	JNK pT183/Y185	Bim	Beclin
Bak	Caspase 3	Cleaved-Caspase 7	JNK2
c-Jun pS73	Wee1 pS642	Annexin VII	XBP1
FoxO3a	IAP2	Bid	Mcl1

Table S5. Combinational effect of ONC201 with seven targeted kinase inhibitors. CI values <0.10 indicated very strong synergism; 0.10–0.30, strong synergism; 0.31–0.70, synergism; 0.71–0.85, moderate synergism; 0.86–0.90, slight synergism; 0.91–1.10, nearly additive; 1.11–1.20, slight antagonism; 1.21–1.45, moderate antagonism; 1.46–3.30, antagonism; 3.31–10, strong antagonism; >10, very strong antagonism. Fa, fractional cell killing effect of drug treatment.

ONC201/Trametinib

Combination (μM)		MDA-MB-231	
ONC201	Trametinib	Fa	CI
0.27	0.32	0.41	1.25
0.54	0.63	0.79	0.37
1.07	1.25	0.86	0.43
2.13	2.50	0.98	0.14
4.25	5.00	1.00	0.11

Combination (μM)		MDA-MB-453	
ONC201	Trametinib	Fa	CI
0.27	0.32	0.50	0.20
0.54	0.63	0.53	0.36
1.07	1.25	0.81	0.34
2.13	2.50	0.93	0.35
4.25	5.00	0.96	0.53

Combination (μM)		SUM149	
ONC201	Trametinib	Fa	CI
1.07	1.25	0.75	0.22
2.13	2.50	0.97	0.09
4.25	5.00	0.99	0.11
8.50	10.00	0.98	0.27
16.90	20.00	0.98	0.49

Combination (μM)		HCC70	
ONC201	Trametinib	Fa	CI
1.07	1.25	0.42	0.11
2.13	2.50	0.58	0.07
4.25	5.00	0.70	0.07
8.50	10.00	0.74	0.12
16.90	20.00	0.88	0.08

ONC201/Ulixertinib

Combination (μM)		MDA-MB-231	
ONC201	Ulixertinib	Fa	CI
0.27	0.32	0.56	0.91
0.54	0.63	0.69	0.95
1.07	1.25	0.78	1.16
2.13	2.50	0.94	0.49
4.25	5.00	0.97	0.57

Combination (μM)		MDA-MB-453	
ONC201	Ulixertinib	Fa	CI
0.54	0.63	0.46	0.76
1.07	1.25	0.81	0.49
2.13	2.50	0.94	0.40
4.25	5.00	0.95	0.75
8.50	10.00	0.98	0.87

Combination (μM)		SUM149	
ONC201	Ulixertinib	Fa	CI
1.07	1.25	0.74	0.33
2.13	2.50	0.80	0.49
4.25	5.00	0.80	0.97
8.50	10.00	0.90	1.03
16.90	20.00	0.90	2.05

Combination (μM)		HCC70	
ONC201	Ulixertinib	Fa	CI
1.07	1.25	0.18	1.29
2.13	2.50	0.37	0.93
4.25	5.00	0.57	0.78
8.50	10.00	0.96	0.08
16.90	20.00	0.99	0.03

ONC201/VX-11e

Combination (μM)		MDA-MB-453	
ONC201	VX-11e	Fa	CI
0.54	0.63	0.55	0.42
1.07	1.25	0.84	0.34
2.13	2.50	0.97	0.24
4.25	5.00	0.99	0.22
8.50	10.00	1.00	0.18

Combination (μM)		MDA-MB-231	
ONC201	VX-11e	Fa	CI
0.27	0.32	0.47	0.78
0.54	0.63	0.64	0.86
1.07	1.25	0.79	0.91
2.13	2.50	0.96	0.45
4.25	5.00	0.99	0.33

Combination (μM)		SUM149	
ONC201	VX-11e	Fa	CI
1.07	1.25	0.86	0.16
2.13	2.50	0.90	0.24
4.25	5.00	0.90	0.48
8.50	10.00	0.90	0.97
16.90	20.00	0.90	1.93

Combination (μM)		HCC70	
ONC201	VX-11e	Fa	CI
1.07	1.25	0.16	0.89
2.13	2.50	0.42	0.58
4.25	5.00	0.67	0.52
8.50	10.00	0.95	0.17
16.90	20.00	0.99	0.11

ONC201/MK-2206

Combination (µM)		MDA-MB-453	
ONC201	MK-2206	Fa	CI
0.27	0.32	0.64	0.70
0.54	0.63	0.87	0.37
1.07	1.25	0.95	0.30
2.13	2.50	0.98	0.27
4.25	5.00	1.00	0.21

Combination (µM)		MDA-MB-231	
ONC201	MK-2206	Fa	CI
1.07	1.25	0.21	1.23
2.13	2.50	0.91	0.28
4.25	5.00	0.96	0.35
8.50	10.00	0.98	0.47
16.90	20.00	0.99	0.62

Combination (µM)		SUM149	
ONC201	MK-2206	Fa	CI
1.07	1.25	0.61	0.38
2.13	2.50	0.77	0.45
4.25	5.00	0.79	0.81
8.50	10.00	0.99	0.12
16.90	20.00	1.00	0.07

Combination (µM)		HCC70	
ONC201	MK-2206	Fa	CI
0.54	0.63	0.52	0.81
1.07	1.25	0.59	0.82
2.13	2.50	0.73	0.36
4.25	5.00	0.87	0.10
8.50	10.00	0.90	0.11

ONC201/PF04691502

Combination (µM)		MDA-MB-453	
ONC201	PF04391052	Fa	CI
0.27	0.32	0.89	0.44
0.54	0.63	0.89	0.81
1.07	1.25	0.89	1.66
2.13	2.50	0.90	3.07
4.25	5.00	0.90	6.05

Combination (µM)		MDA-MB-231	
ONC201	PF04391052	Fa	CI
0.14	0.16	0.26	2.19
0.27	0.32	0.49	2.08
0.54	0.63	0.69	2.16
1.07	1.25	0.79	2.92
2.13	2.50	0.81	5.33

Combination (µM)		SUM149	
ONC201	PF04391052	Fa	CI
0.27	0.32	0.30	0.49
0.54	0.63	0.31	0.95
1.07	1.25	0.95	0.28
2.13	2.50	0.98	0.33
4.25	5.00	0.98	0.74

Combination (µM)		HCC70	
ONC201	PF04391052	Fa	CI
0.07	0.08	0.37	1.77
0.14	0.16	0.57	1.50
0.27	0.32	0.75	1.24
0.54	0.63	0.87	1.00
1.07	1.25	0.90	1.41

ONC201/Buparlisib

Combination		MDA-MB-453	
ONC201	Buparlisib	Fa	CI
0.07	0.08	0.30	0.41
0.14	0.16	0.40	0.59
0.27	0.32	0.50	0.87
0.54	0.63	0.58	1.37
1.07	1.25	0.90	0.68

Combination		MDA-MB-231	
ONC201	Buparlisib	Fa	CI
0.54	0.63	0.36	2.63
1.07	1.25	0.64	2.27
2.13	2.50	0.83	2.15
4.25	5.00	0.84	3.98
8.50	10.00	0.85	7.71

Combination		SUM149	
ONC201	Buparlisib	Fa	CI
1.07	1.25	0.91	0.28
2.13	2.50	0.96	0.32
4.25	5.00	0.98	0.50
8.50	10.00	0.99	0.78
16.90	20.00	1.00	0.67

Combination		HCC70	
ONC201	Buparlisib	Fa	CI
0.27	0.32	0.30	0.90
0.54	0.63	0.31	1.74
1.07	1.25	0.87	0.49
2.13	2.50	0.87	1.00
4.25	5.00	0.92	1.32

ONC201/Dacotlisib

Combination		MDA-MB-453	
ONC201	Dactolisib	Fa	CI
0.14	0.16	0.64	48.17
0.27	0.32	0.75	15.26
0.54	0.63	0.77	18.02
1.07	1.25	0.77	38.72
2.13	2.50	0.77	72.98

Combination		MDA-MB-231	
ONC201	Dactolisib	Fa	CI
0.27	0.32	0.42	3299.18
0.54	0.63	0.53	1063.83
1.07	1.25	0.63	504.16
2.13	2.50	0.79	43.43
4.25	5.00	0.86	15.03

Combination		SUM149	
ONC201	Dactolisib	Fa	CI
0.14	0.16	0.30	2.20
0.27	0.32	0.40	1.97
0.54	0.63	0.50	1.89
1.07	1.25	0.54	2.89
2.13	2.50	0.75	1.29

Combination		HCC70	
ONC201	Dactolisib	Fa	CI
1.07	1.25	0.10	133.16
2.13	2.50	0.19	101.41
4.25	5.00	0.57	20.76
8.50	10.00	0.74	15.65
16.90	20.00	0.76	27.63

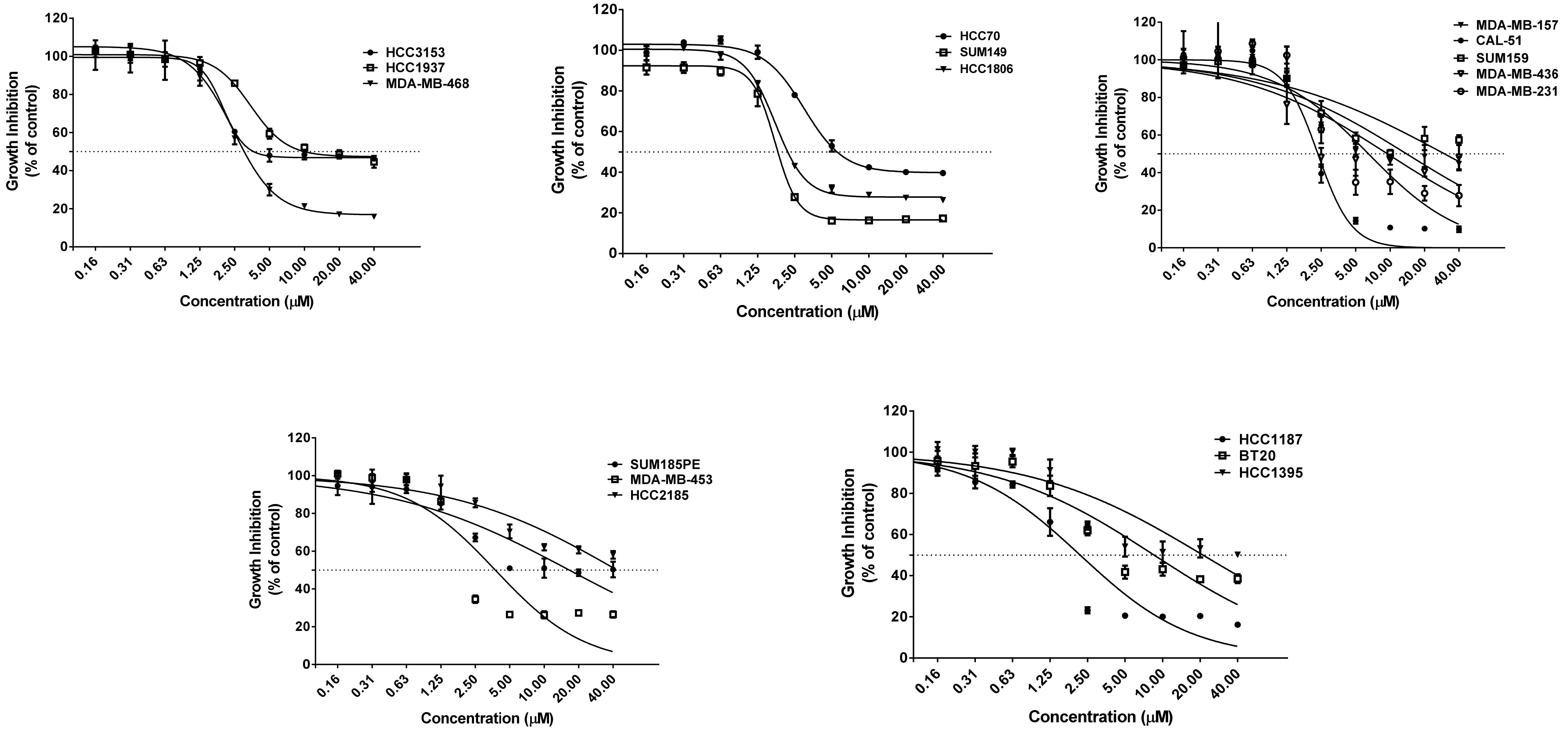

Figure S1. Dose-response of ONC201 in TNBC cell lines with Vanderbilt TNBC molecular subtypes. TNBC cells were treated with ONC-201 for 5 days, and viability was measured by using sulforhodamine-B assays. Data shown are representative of three experiments with similar results.

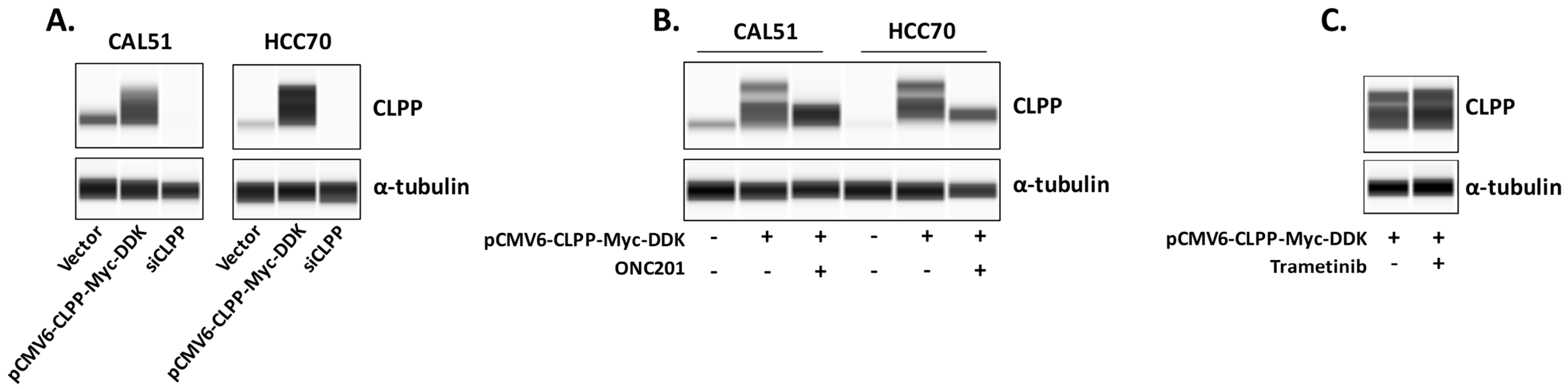

Figure S2. Western blotting. CAL51 and HCC70 cells were transfected with siClpP (SASI_Hs01_00025718, Sigma-Aldrich, USA), pCMV6-ClpP-Myc-DDK or control vectors (OriGene, USA) by Neon electroporation transfection kit (Invitrogen, USA). (**A**) Overexpression or knockdown of ClpP by expression vector or siRNA. (**B**) ONC-201 reduced ClpP expression in ClpP-overexpressed TNBC cell lines. (**C**) Trametinib does not affect on ClpP expression.
